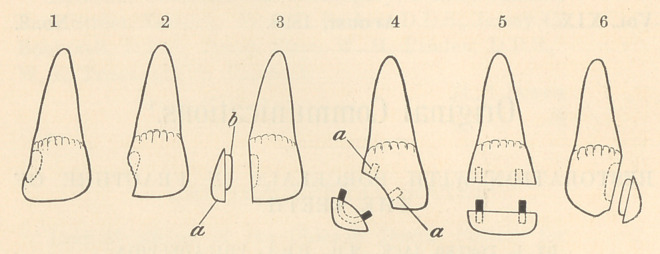# Restoration with Porcelain in Fracture of the Teeth

**Published:** 1898-08

**Authors:** L. Foster Jack

**Affiliations:** Philadelphia


### International Dental Journal.

VOL. XIX. AUGUST, 1898. No. 8.

### Original Communications.[1]

### RESTORATION WITH PORCELAIN IN FRACTURE OF THE TEETH.[2]

BY L. FOSTER JACK, M.D., D.D.S., PHILADELPHIA.

I PRESENT a few notes and a diagram or two from which I hope to demonstrate the use of porcelains for the restoration of teeth that have been fractured. The method to be described is particularly directed in its application to those cases wherein the vitality of the tooth has not been disturbed,—that is, where the pulp has been but slightly or not at all encroached upon.

The use of porcelains is especially called for in fractures occurring in the superior incisors, but they may be useful as far back as the anterior surface of the first molar.

Pre-eminent stands the central incisor, most subject to fracture, either by a blow, a fall, or in ordinary use.

To be brief, we will turn to the diagram (Fig. 1). Here we have a left central weakened by decay on its mesial surface. The enamel, having been robbed of its support, is weakened and becomes frail. Its possessor bites upon something unusually hard, breaking away the corner, leaving an unsightly gap. (Fig. 2.) To

[1] The editor and publishers are not responsible for the views of authors of papers published in this department, nor for any claim to novelty, or otherwise, that may be made by them. No papers will be received for this department that have appeared in any other journal published in the country.

[2] Read before the Academy of Stomatology, March 22, 1898.

remedy this defect now becomes our task. It is simple but tedious, for the parts are small, and it requires exactness to be rewarded with a good result.

The first step in the operation is to cut the irregular walls formed by the fracture and cavity in a direct line from the cutting edge to the cervix (Fig. 3), grinding at the same time both the labial and lingual walls to the same plane, thus making a flat and even surface, with the exception of the cavity in the upper central portion. This is accomplished with a corundum or carborundum disk, one side of which is flat.

The next step is the preparation of the cavity. After the removal of all decay and softened tooth-structure the cavity is extended as far towards the cutting edge of the tooth as is compatible with strength, but is not made deep, care being exercised to avoid the pulp. The edges of the cavity are but slightly undercut. The floor should be nearly flat and convex if encroachment upon the pulp is feared. If the pulp is in danger, it should be protected from pressure, which may be exerted at a subsequent stage of the operation.

The tooth being now prepared, we proceed to the formation of the porcelain counterpart. The cavity in the tooth is lined with platinum foil; this is carefully removed, filled with porcelain body, and the latter fused in the Downie furnace. After stripping off the platinum the porcelain body is then placed in the cavity and ground flush with the walls.

An all-porcelain tooth-crown of appropriate color, and corresponding in form and size as nearly to the natural tooth as possible, is selected. From this we cut with a disk that portion which is desired for substitution. This is ground on the lathe until it is sufficiently diminished in size to correspond to the absent part of the tooth.

The grinding is done principally upon the straight side, and it is not necessary to cut the labial surface if the tooth-crown has been well selected.

The relation of the outer porcelain (*a*) and inner, or retaining porcelain (*b*), is obtained by temporarily fastening the two together with wax and fitting them to the tooth. The wax is then replaced by a thinly mixed layer of the porcelain body and the two parts are fused together in the furnace. The piece is cemented to place, finely fitted, and polished.

Fig. 4 represents a case of fracture of the distal portion of a right central incisor, one which would probably only occur in a child from the result of a fall. In this case the preparation of the tooth is a simple operation. The line of fracture is made free from all irregularity and the curve trued. Two pits are drilled at the points *a*, *a* to the depth of one-eighth of an inch, in diameter large enough to freely admit No. 19 standard wire. It may be found necessary to enlarge them laterally at the opening to receive the retaining posts. The substitute is selected and cut from a tooth-crown as described in the foregoing case. It is then deeply grooved, with a diamond-shod disk, from the contact surface inward, forming an opening semicircular in form. Into this a piece of No. 19 stiff platinum wire is fitted, the ends being allowed to protrude one-eighth of an inch. The groove is then filled with porcelain body and fused. It is then ready to be cemented into place.

Fig. 5 indicates a straight fracture involving the incisal third of a central or, more frequently, a lateral incisor. The procedure in the preparation of the stump is the same as in the case preceding.

The porcelain tip is also formed as in the last operation, with the exception of the insertion of the retaining posts.

For the case under consideration a diamond-pointed drill becomes requisite, for the purpose of forming two pits for the reception of the retaining posts. These are of platinum and are retained by fusing as was the wire in the previous case.

Bicuspids and molars, in which the mesial and a portion of the buccal surface has been lost (Fig. 6), can be restored in the way described for the proximate surfaces of the incisors.

It must be evident to all that the chief advantage of the method, if it has any, is in the fact that the porcelains can be ground to fit perfectly without the hinderance of pins or posts. Also that the porcelains are not baked for individual cases, but are selected from tooth-crowns in stock. Thus obviating the uncertainty in color. The method enabling one to select a shade and form as nearly perfect as possible.

Crowns best suited for the purpose are those of a fine texture, as they can be ground and polished to fit any irregularity and because there is less color-change than in the more porous bodies. By this I mean that there is often a surprising change manifested in the color of a porcelain after it has been set, due probably to the reflection and absorption of light. In this connection the selection of cement is an important consideration, for the porcelain can be varied a degree or two in shade, either lighter or darker.

## Figures and Tables

**Figure f1:**